# Integrative Bone Metabolomics—Lipidomics Strategy for Pathological Mechanism of Postmenopausal Osteoporosis Mouse Model

**DOI:** 10.1038/s41598-018-34574-6

**Published:** 2018-11-07

**Authors:** Hongxia Zhao, Xiaoqun Li, Dianying Zhang, Haiyan Chen, Yufan Chao, Kaiwen Wu, Xin Dong, Jiacan Su

**Affiliations:** 1 Institute of translational medicine, Shanghai University, Shanghai, 200444 China; 2 Graduate Management Unit, Shanghai Changhai Hospital, Second Military Medical University, Shanghai, 200433 China; 3 Department of Orthopedics Trauma, Peking University People’s Hospital, Beijing, 100009 China; 4 Zhongshan Hospital of Fudan University, 180 Fenglin Road, Shanghai, 200032 China; 5 School of Pharmacy, Second Military Medical University, Shanghai, 200433 China; 6 Nine Company, Faculty of Medicine, Second Military Medical University, Shanghai, 200120 China; 7 Department of Orthopedics Trauma, Shanghai Changhai Hospital, Second Military Medical University, Shanghai, 200433 China

## Abstract

Osteoporosis, characterized by bone mass reduction and increased fractures, has become a global health problem that seriously affects the health of people, especially postmenopausal women; however, the current pathogenesis of postmenopausal osteoporosis (PMOP) has not been thoroughly elucidated to date. In this study, bilateral ovariectomy was performed to establish an OVX mouse model of osteoporosis. UPLC-Q-TOF-MS-based lipidomics in combination with metabolomics were used to analyze the femur tissue of osteoporosis mice. We found that 11 polar metabolites and 93 lipid metabolites were significantly changed and were involved in amino acid metabolism, nucleotide metabolism and lipid metabolism. Among the lipids, fatty acyls, glycerolipids, glycerophospholipids, sphingolipids and sterols showed robust changes. These results revealed that several metabolic disorders caused by changes in the hormone levels in OVX, especially disordered lipid metabolism, are closely related to the imbalance between bone resorption and formation and may underlie the development of PMOP. The data generated via lipidomics and metabolomics presented in this study shows good applicability and wide coverage in the construction of the metabolic profile of bone tissue. Therefore, this approach may provide the pathway focusing and data support at the metabolite level for the in-depth mechanism of PMOP.

## Introduction

Osteoporosis is a progressive, age-related metabolic bone disease, it characterized by generalized reduced bone mass that is accompanied by microstructural degeneration of bone tissue, resulting in decreased bone mineral density (BMD) and increased risk of fracture^1,2^. Osteoporosis can be divided into primary and secondary, with postmenopausal osteoporosis (PMOP) being the most common primary form^3^. With aging of worldwide population, osteoporosis has become a global health problem that seriously affects the health of middle-aged and aged people, especially in middle-aged and aged women, which burdens the government financial and healthcare system greatly^4^.

The imbalance between bone resorption and formation is the pathophysiological basis of osteoporosis^5^. The current studies of osteoporosis mainly primarily focus on the differentiation, regulation and balance of osteoblasts and osteoclasts^6^. Many factors can play a role in PMOP formation, including metabolic disorders, especially hormone imbalance^3^. Thus, studies of the internal metabolic environment of PMOP to monitor changes in endogenous metabolites related bone resorption and bone formation disorders will help to determine the mechanisms of osteoporosis.

Metabolomics, a strategy to study endogenous small-molecule metabolites systematically, can explore changes in the metabolic environment after the dynamic stimulation of pathophysiological, environmental or genetic factors^7^. Furthermore, metabolomics has been widely used in exploration of pathologic mechanism, discovery of biomarker and toxicity and safety study of drugs^8,9^. As the object of metabolomics studies is to quantify downstream molecules of the bioinformatics flow, which is close to biological phenotypes^10^, metabolomics can more readily screen out phenotype-related key biological nodes or biological pathways for diseases with complex causes and unknown mechanisms.

Recently, the application of metabolomics for the study of osteoporosis has attracted more and more attention^11,12^; however, most of these early studies used patients’ peripheral body fluids, including serum, plasma, and urine^13–15^, and bone tissue-related literature is notably rare. Metabolites changes in the peripheral body fluids include metabolic changes not only induced by osteoporosis but also induced by outside stimuli. So many confounding factors made it hard to indicate the pathogenesis directly and clearly. Bone tissue, the target tissue of osteoporosis, can be used to uncover the metabolic dysregulation directly associated with the development of osteoporosis. Additionally, due to the failure to use more advanced and targeted methods, the current bone metabolomic studies of osteoporosis reflect an incomplete characterization of metabolic changes. Certain metabolic molecules with important biological functions, including lipids, were omitted from these previous studies. Lipids play an important role in cell function maintenance and energy storage^16^ and have a close relationship with osteoporosis^17–19^. The traditional metabolomics studies have limitations in the extraction and analysis of lipid molecules, which greatly influenced the characterization of lipids^20^. In recent years, lipidomics has been developed to analyze lipid molecules in a more targeted fashion, especially those based on electrospray ionization mass spectrometry (ESI-MS), which has been widely used in lipid metabolism, lipid mediated signal transduction and other related fields^21,22^. Therefore, the application of advanced techniques in metabolomics can provide more complete and more accurate data for the interpretation of osteoporosis-related metabolic changes.

As mice can reproduce very similar models of human diseases as well as clear genetic background, *in vivo* microorganism controlled, stable and cheap, lots of researchers preferred establishing mice model to understand human clinical diseases^23,24^. So in our study, an ovariectomized (OVX) mouse model was established to simulate postmenopausal osteoporosis^25^. Lipidomics in combination with metabolomics analyses were performed on the femur to generate a bone metabolism profile of PMOP. To demonstrate the differential metabolites and metabolic pathways involved in the OVX model, we provide the complete metabolic data and a clear direction for revealing the metabolic dysregulation and mechanisms associated with PMOP occurrence and development.

## Result

### Micro-CT analysis and histological analysis

**Figure 1:**
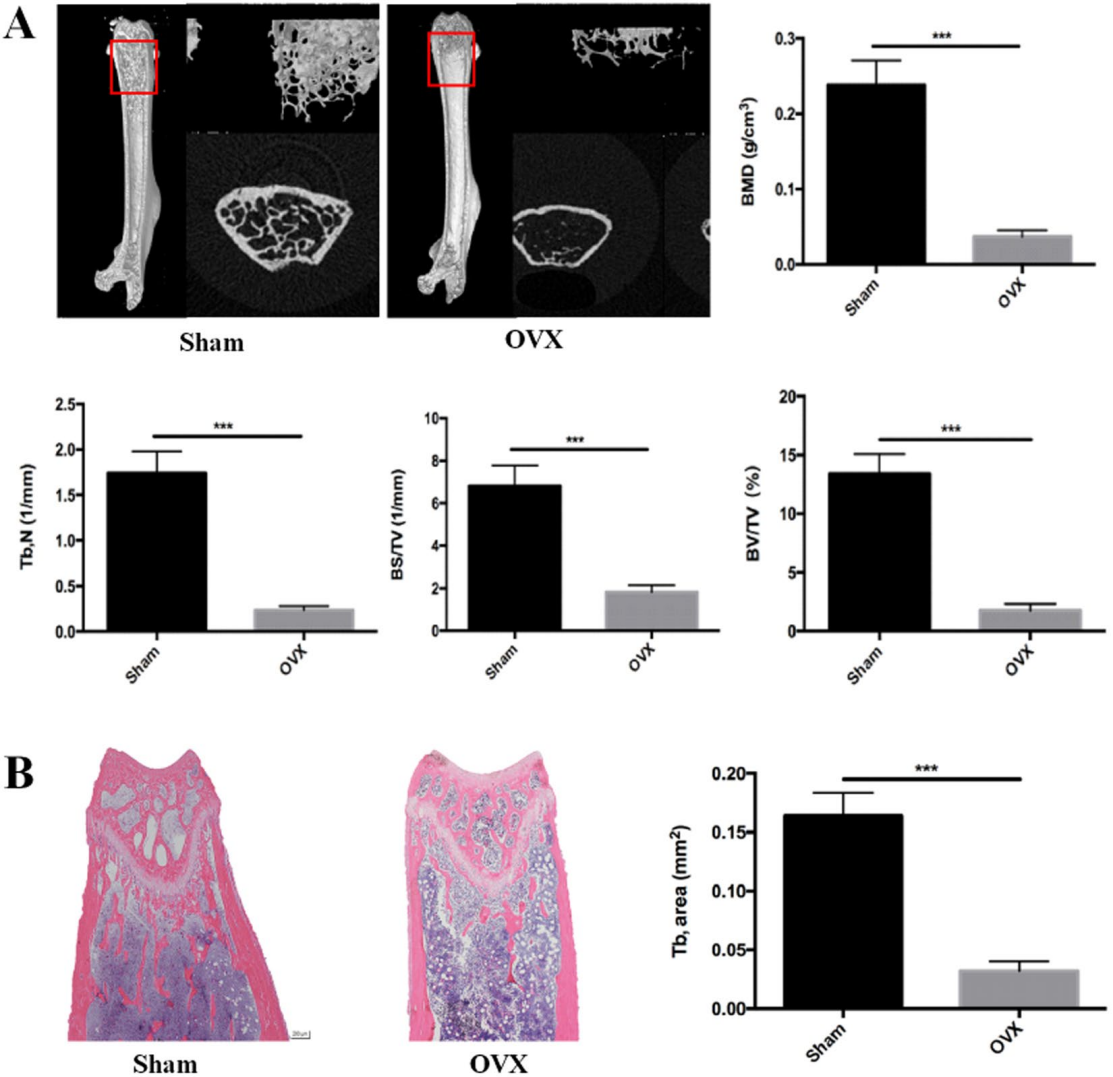
Ovariectomy-induced bone loss *in vivo*. (**A**) Micro CT analysis of the distal femur from sham and OVX group. Calculations of bone mineral density (BMD), trabecular number (Tb, N), bone surface area/ total value (BS/TV), bone value/total value (BV/TV). (**B**) H&E staining of distal femoral sections and quantification of trabecular area from sham and OVX group. (***P < 0.001).

Micro-CT and histological analysis are taken to show bone loss after an OVX operation. In Fig. 1A, Micro-CT with 3D reconstruction of the femur shows that BMD (g/cm3), trabecular number (Tb.N, 1/mm), bone surface area/total value (BS/TV, 1/mm) and bone value/total value (BV/TV, %) were reduced in OVX group (P < 0.001) compared to the sham group, all four parameters revealed significant bone loss. Further histological and histomorphometric analyses confirmed the destruction on bone after OVX operation (Fig. 1B). Less bone trabecular was observed in the OVX group, revealing significantly bone loss and impaired trabecular microarchitecture in the femur bone. All these results suggested that we established a successful osteoporosis model.

### Metabolic and lipidomic profiling analysis of femur

**Figure 2:**
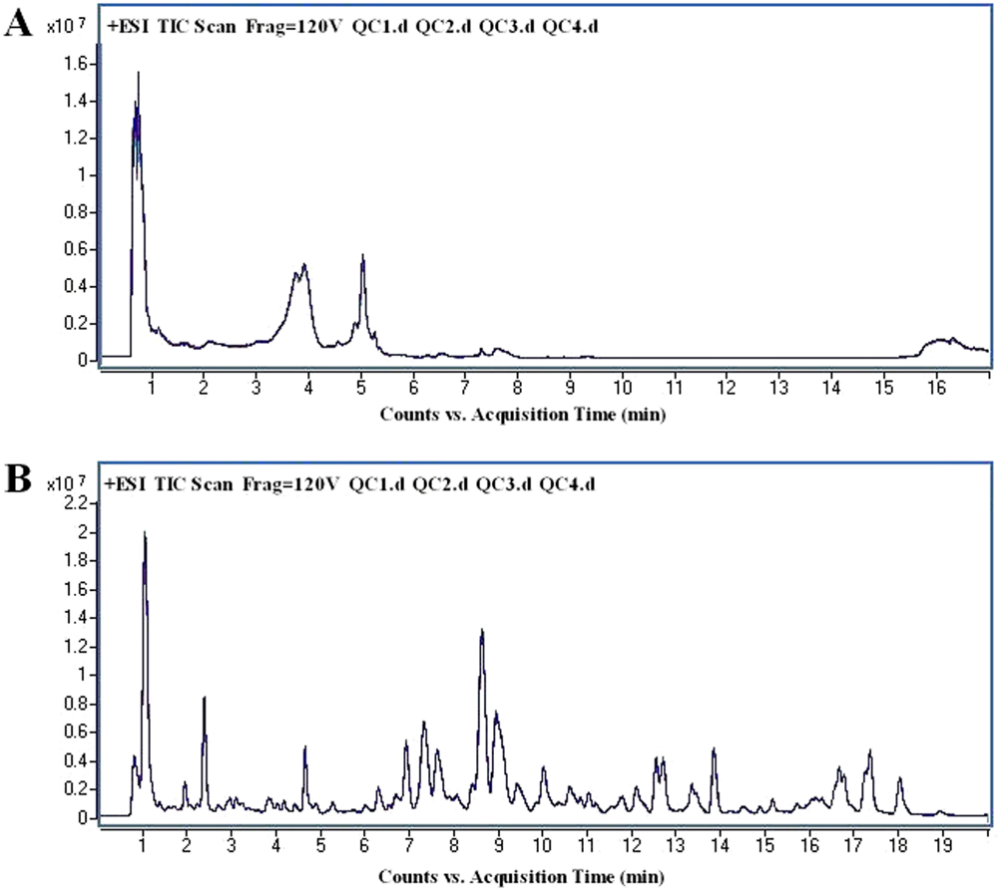
The overlapped total ion chromatography (TIC) of 4 QC samples in positive ion mode in both (**A**) metabolomic analysis and (**B**) lipidomic analysis.

**Figure 3:**
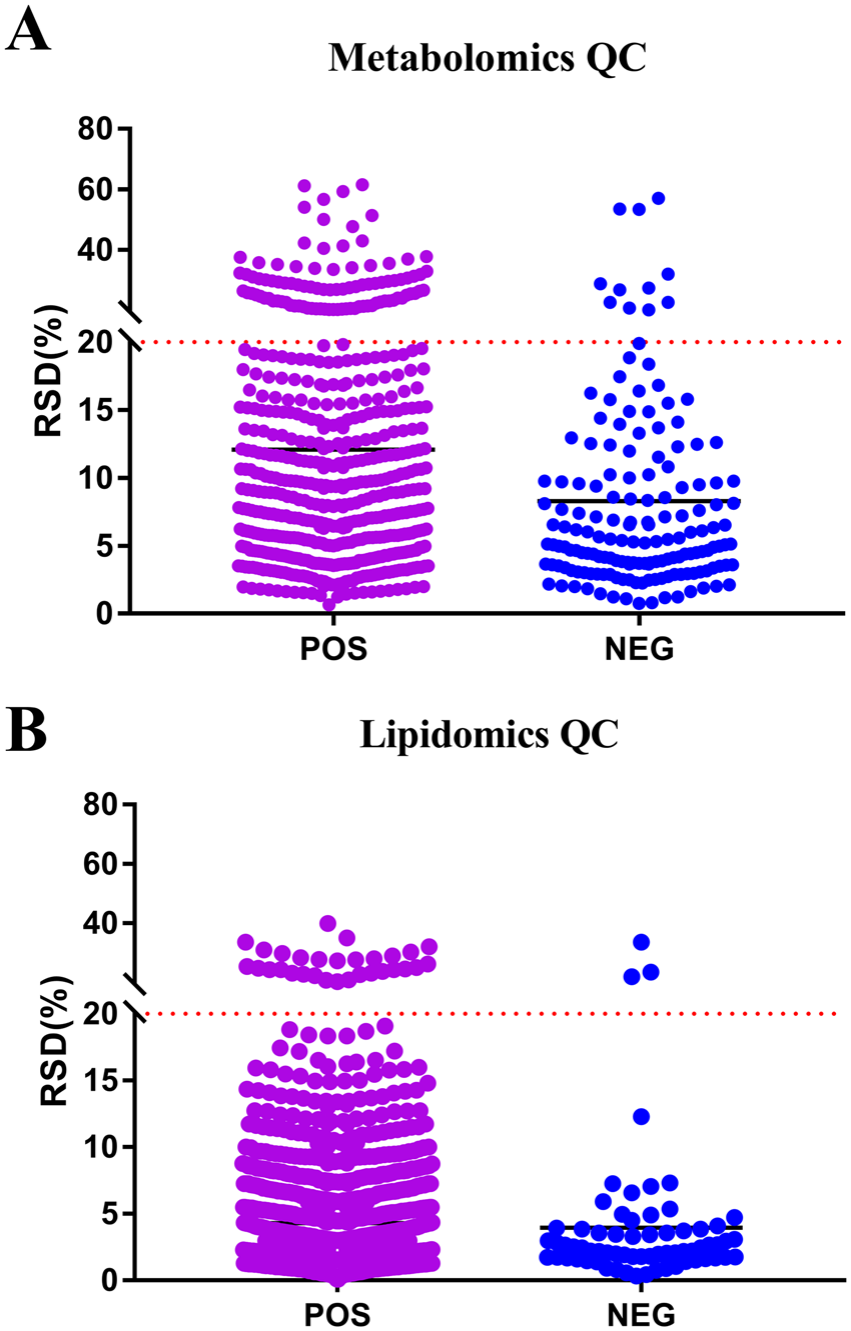
The RSD values of the peak intensities in QC samples. (**A**) Scatter plot of RSD values in metabolomics QC samples (**B**) Scatter plot of RSD values in lipidomics QC samples.

The system stability was carried out by injecting a QC sample every 4 samples during the whole sample batch. The total ion chromatography (TIC) the 4 QC samples displayed in Fig. 2A,B showed a good overlap in positive mode. Negative mode data is shown in Supplementary Figure S1A,B. The RSD values of the peak intensities in the QC samples were measured for stability. As is shown in Fig. 3, more than 80% of the RSD values of the QC samples in metabolomics and lipidomics analysis were less than 20%. These results demonstrated that the stability of the proposed method was sufficient.

**Figure 4:**
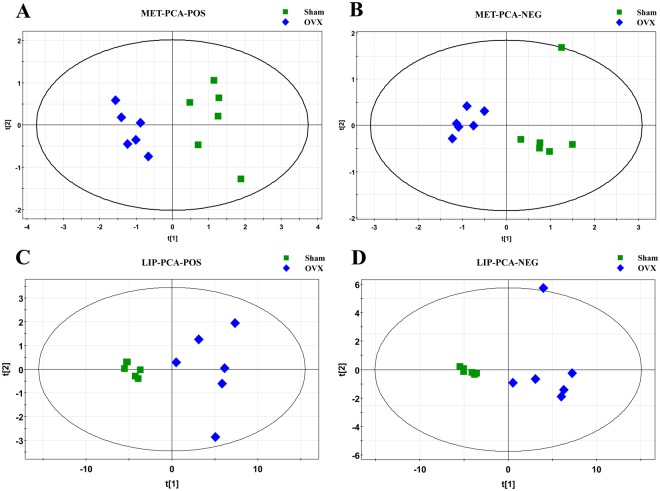
PCA score plot of multivariate statistical analysis of the experimental group in ESI positive and negative ion mode. (**A**,**B**) PCA score plot of the sham and OVX group in ESI positive and negative ion mode respectively in metabolomic analysis; (**C**,**D**) PCA score plot of the sham and OVX group in ESI positive and negative ion mode respectively in lipidomic analysis.

**Figure 5:**
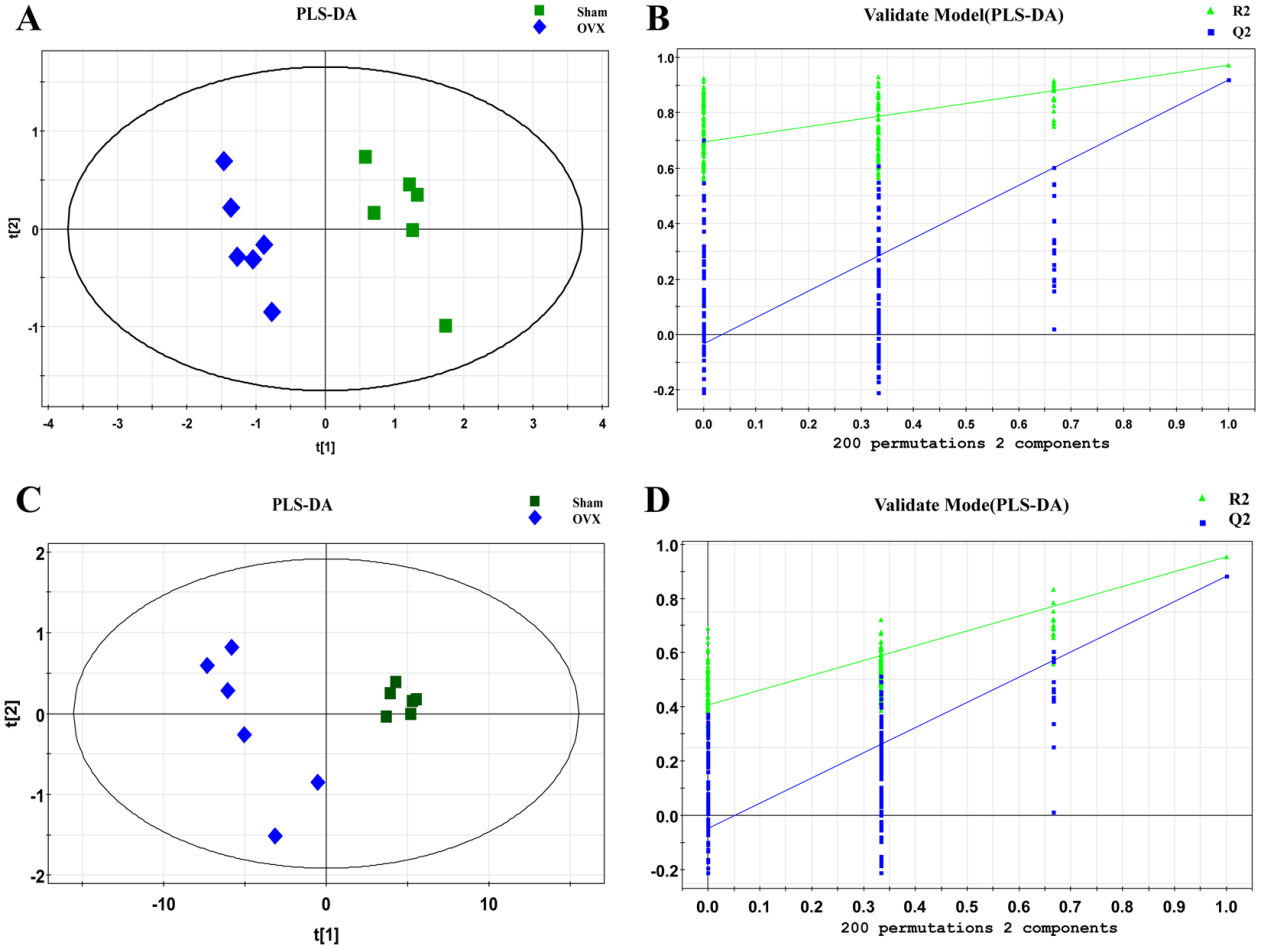
Plots of multivariate statistical analysis of the experimental group in ESI positive ion mode.(**A**,**C**) PLS-DA score plot of the sham and OVX group in metabolomic and lipidomic analysis respectively; (**B**,**D**) Permutation test plot of the sham and OVX group in metabolomic and lipidomic analysis respectively.

**Figure 6:**
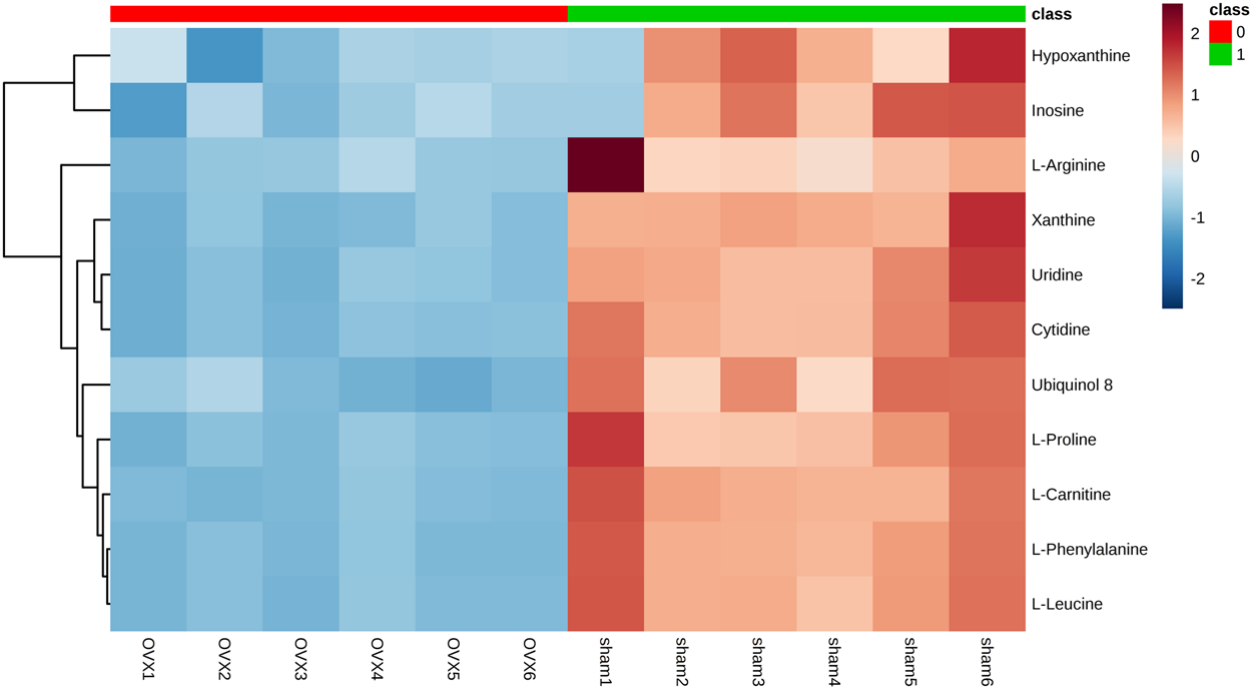
Heat map based on the relative levels of polar metabolites in femur of mouse in OVX. Class 0: OVX group, Class 1: Sham. Color key indicates metabolite expression value, red: up-regulated, blue: down-regulated.

The entire normalized data from all samples was imported into SIMCA-P program (version 11) to perform principal components analysis (PCA) and partial least square discriminate analysis (PLS-DA). PCA, an unsupervised method, is applied as the first step in the separation procedure to reduce the dimension of data and make the observation more straightforward; PLS-DA, a supervised method that has a similar principle to PCA, is used to enhance the classification performance^26,27^. The PCA score plots in both modes showed that there was no outlier and that the Sham group was clearly separated from the OVX group in both the metabolic and lipidomic analyses (Fig. 4). Both modes of the supervised PLS-DA scores plots (Fig. 5A,C and Supplementary Figure S2A,C) displayed clearly separated clusters between the two groups. The reliability of the established PLS-DA models was evaluated by the explained variation R2 and the predicted total variation Q2, which is calculated by cross-validation. The expected R2 and Q2, highly dependent on their application model, should be more than 0.5 and 0.4, respectively, for a significant biological model^28^. In our established model for PMOP, the value of R2 and Q2 were above 0.6 (Table 1), indicating that the PLS-DA model was established successfully. R2 and Q2 from the PLS-DA analyses were calculated in the permutation test and shown in Fig. 5B,D and Supplementary Figure S2B,D. In the present research, ions with VIPs greater than 1.0 were considered to be important differential metabolites. Students t-test was performed to assess the statistical significance. Finally, 11 metabolites (Table 2, all decreased in the OVX group) and 93 lipids (Supplementary Table S1, 9 decreased and 84 increased in the OVX group) with significant differences between the sham and OVX groups were identified as potential biomarkers. The heat map (Fig. 6 and Supplementary Figure S3) of potential biomarkers was performed using the MetaboAnalyst platform (http://www.metaboanalyst.ca) to visually display the differences between groups.

**Table 1:** Summary of the parameters for assessing quality of partial least square discriminate analysis (PLS-DA) model.

OVX-Sham	Mode	No. Component	R2X	R2Y	Q2
Metabolic Analysis	Positive	2	0.652	0.965	0.906
	Negative	2	0.698	0.966	0.892
Lipidomic Analysis	Positive	2	0.895	0.952	0.883
	Negative	2	0.926	0.903	0.855

**Table 2:** Differential metabolites expressed in OVX group.

No.	Name	Formula	m/z	Adduct	Rt(min)	FC(M-C)	P(M-C)	FDR	Pathway
1	Uridine^a^	C_9_H_12_N_2_O_6_	243.0607	[M − H]^−^	3.61	0.49↓	2.83E-08***	4.143E-07	Pyrimidine metabolism
2	Hypoxanthine^b^	C_5_H_4_N_4_O	137.0458	[M + H]^+^	3.73	0.78↓	3.13E-04***	6.808E-07	Purine metabolism
3	Ubiquinol 8^c^	C_49_H_74_O_4_	744.5893	[M + NH4]^+^	3.93	0.44↓	2.35E-08***	1.785E-05	Respiratory chain
4	Xanthine^b^	C_5_H_4_N_4_O_2_	151.0246	[M − H]^−^	4.58	0.61↓	7.07E-08***	8.762E-07	Purine metabolism
5	Inosine^b^	C_10_H_12_N_4_O_5_	267.0722	[M − H]^−^	5.16	0.60↓	6.77E-03**	1.515E-02	Purine metabolism
6	Cytidine^a^	C_9_H_13_N_3_O_5_	242.0768	[M − H]^−^	6.25	0.31↓	6.09E-09***	1.963E-07	Pyrimidine metabolism
	Cytidine^a^	C_9_H_13_N_3_O_5_	266.0750	[M + Na] +	6.27	0.36↓	5.44E-09***	9.595E-05	Pyrimidine metabolism
7	L-Phenylalanine^b^	C_9_H_11_NO_2_	166.0862	[M + H]^+^	7.20	0.27↓	5.73E-09***	7.061E-07	Phenylalanine metabolism
8	L-Leucine^b^	C_6_H_13_NO_2_	132.1019	[M+H]^+^	7.21	0.29↓	6.00E-09***	9.846E-05	Leucine and isoleucine metabolism
9	L-Carnitine^a^	C_7_H_15_NO_3_	162.1126	[M + H]^+^	7.31	0.35↓	5.31E-09***	1.385E-04	Carnitine metabolism
10	L-Proline^b^	C_5_H_9_NO_2_	116.0707	[M + H]^+^	9.35	0.35↓	2.04E-08***	2.927E-03	Arginine and proline metabolism
11	L-Arginine^b^	C_6_H_14_N_4_O_2_	175.1191	[M + H]^+^	16.71	0.41↓	1.40E-05***	1.333E-03	Arginine and proline metabolism

^a^Metabolites analyzed based on MS/MS chromatograms.

^b^Metabolites validated with standards.

^c^Metabolites putatively annotated.

(↓): down-regulated. **p < 0.01, ***p < 0.001 versus Sham.

### Biomarker identification and pathways influenced by OVX in the mouse femur

**Figure 7:**
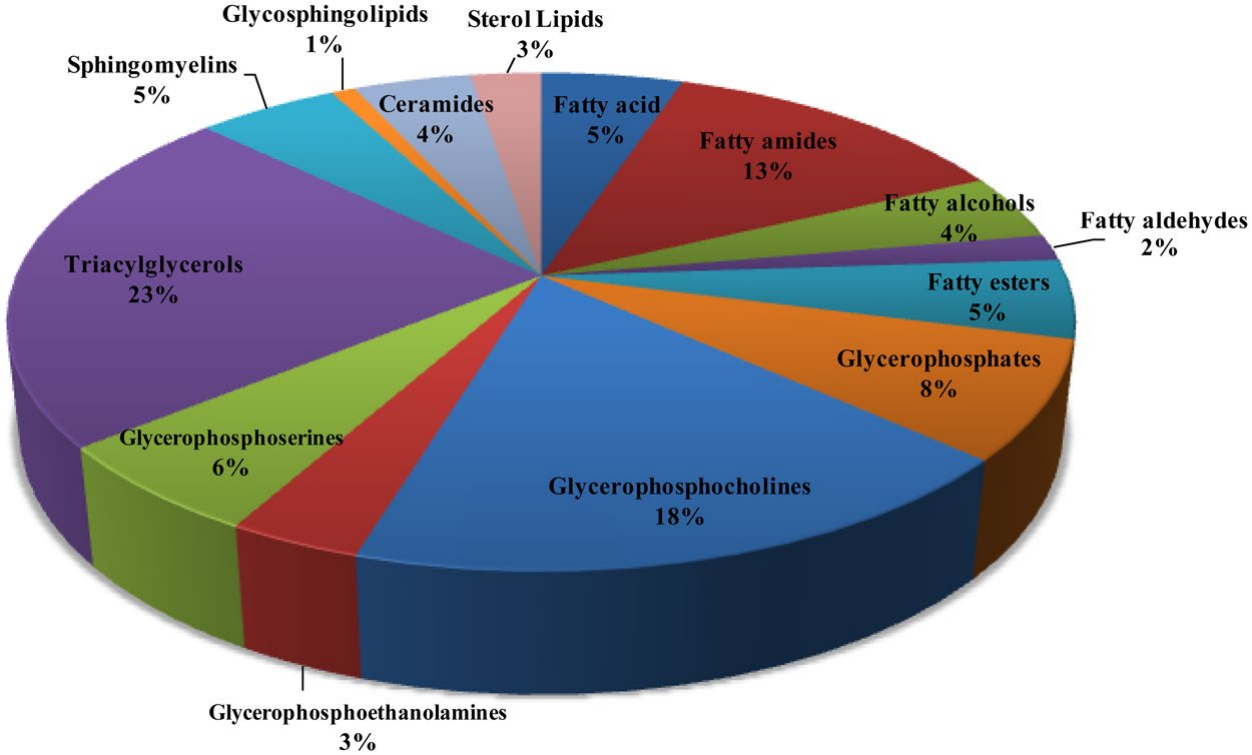
The proportion of different lipid categories.

**Figure 8:**
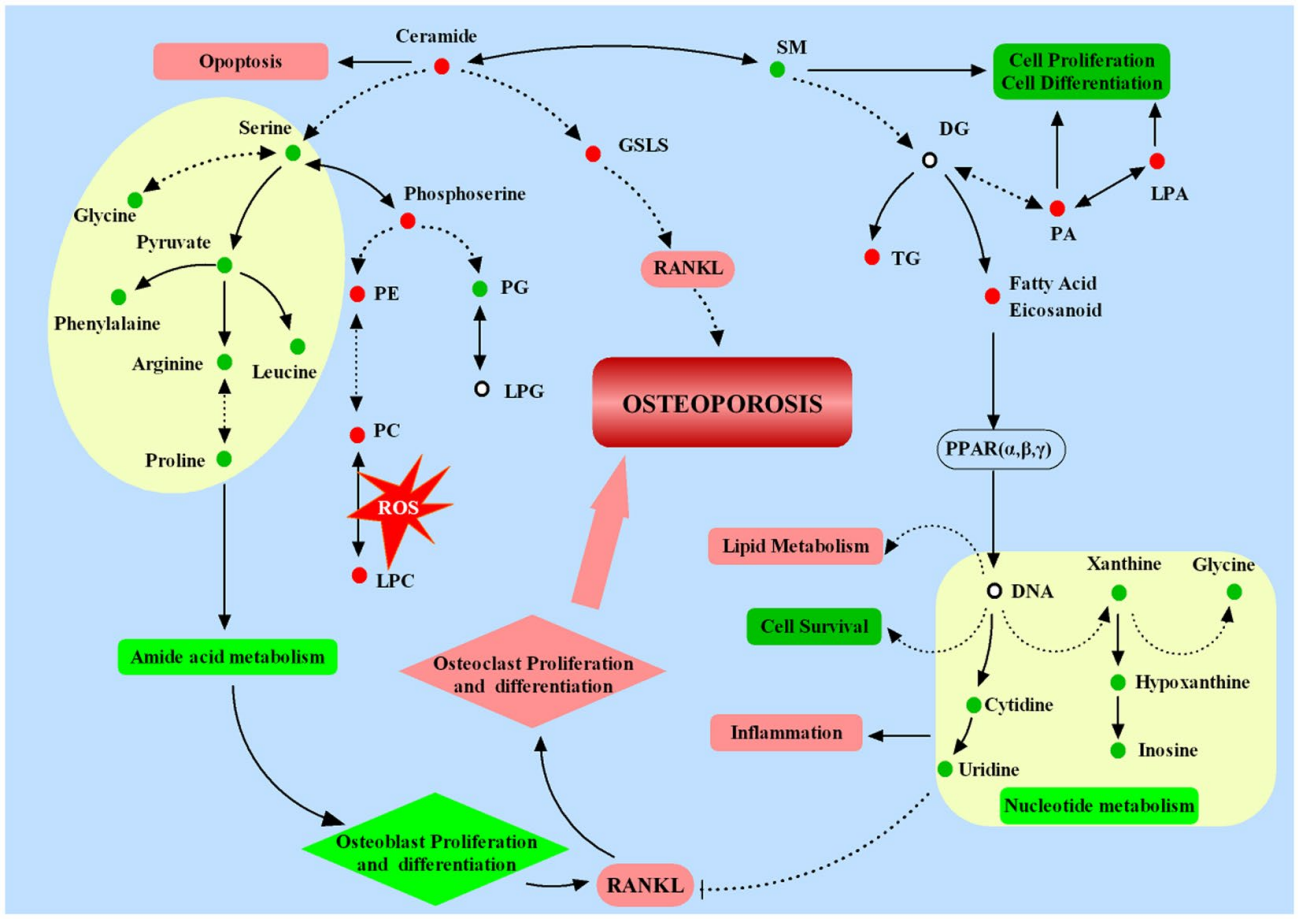
Molecular profiling showing the proposed mechanisms underlying OVX mouse model. : Increased in OVX group compared with shame group; : Decreased in OVX group compared with shame group. Solid line: direct connection, Dotted line: indirect connection. →: transform or promote, ⊥: Inhibit.

Potential biomarkers were identified using the method mentioned above. We categorized the potential biomarkers based on KEGG (http://www.kegg.jp/) and found that the identified biomarkers are mainly in the amino acid, purine, and pyrimidine classes. Lipids were categorized into 14 subclasses, including fatty acyls, glycerophospholipids (PG, PA, PC, PE, PS), triacylglycerols (TG), sphingolipids (SM, Cer) and sterol lipids. The proportion of different lipid categories is shown in Fig. 7. The classification indicated that in established osteoporosis, the femur has multiple changes in several metabolic pathways and function (Fig. 8).

## Discussion

The OVX mouse model is a classical model of PMOP^25^. In this study, lipidomics in combination with polar metabolomics were performed to construct a comprehensive metabolic profile of the femur in OVX-induced osteoporotic mice and showed comprehensive changes in lipid metabolites to polar metabolites. The results suggested that some endogenous metabolites of the bone change significantly during the initiation and progression of PMOP in this mouse model. The polar metabolites included amino acids, purines and pyrimidine nucleotides. The lipid metabolites included fatty acyls, glycerolipids, glycerophospholipids, sphingolipids and sterols. These lipid metabolites and polar metabolites are involved in multiple metabolic pathways, and interact with each other to form a complex network. As is shown in Fig. 8, amino acid and nucleotide metabolism were down-regulated, while lipid metabolism was up-regulated. These disorders not only reduced osteoblast but also increased osteoclast proliferation and differentiation, and increased ROS, as well as inflammation, leading to the development of osteoporosis eventually. This complex network help to explain the mechanism and metabolisms underlying the metabolic disorders associated with PMOP.

Amino acids play an important role in the process of bone metabolism. Arginine, leucine, proline and phenylalanine are all able to promote insulin secretion and enhance the effects of insulin-like growth factor 1 (IGF-1)^29,30^. IGF-1 is capable of promoting the proliferation and differentiation of osteoblasts^31^. Furthermore, amino acids, such as proline, phenylalanine and its analogues, were reported to inhibit osteoclastogenesis and decrease bone resorption in osteoporosis^32,33^. Thus, concentration changes of these amino acids are likely to cause an imbalance of bone metabolism. Meanwhile, post-menopausal osteoporosis does exist in the lack of some amino acids, and increasing the intake of some amino acids could increase BMD and decrease the risk of incidence of osteoporosis^34^. Chevalley *et al*. reported that arginine is able to increase growth hormone secretion and insulin-like growth factor-I production^35^. Fujita *et al*. discovered that leucine has a direct effect on the initiation of mRNA translation, participates in the synthesis of proteins, and maintains sufficient bone strength and bone density^36^. Phenylalanine is reported closely associated with body growth and normal physiological function^37^. In our study, we found that the above amino acids were significantly reduced in bone tissue of PMOP, suggesting that metabolism of amino acids is nonnegligible in the occurrence and development of osteoporosis. In addition, among the nucleotide substances, xanthine, hypoxanthine and inosine are present in purine metabolic pathways. Uridine and cytidine are present in pyrimidine metabolism pathway. According to precious studies, pyrimidine and its derivatives and analogs not only have anti-inflammatory and anti-oxidation effects^38,39^, but also can inhibit osteoclast genesis and bone resorption by inhibiting RANKL^40^. The abnormalities of these pathways found in our study are consistent with these early studies and suggests that the dysfunction of nucleotide occurrs in osteoporosis as well.

Through lipidomics analysis, we found that the occurrence of osteoporosis in the femur of mice was associated with many changes in lipids, including fatty acyls, glycerolipids, glycerophospholipids, sphingolipids and sterols. Fatty acids are a very important subclass of fatty acyls. Among the altered metabolites in our study, we found many fatty acids with a consistent uptrend in the bone tissue of the OVX group compared with sham animals. A previous study revealed that the concentration of cholesterol and fatty acids were increased in PMOP^41^. Zhu *et al*. also found that fatty acids increased in ovariectomy-induced group using metabolomics based on UPLC-Q-TOF-MS^13^. Moreover, fatty amines, fatty alcohols and fatty esters all belong to the fatty acyl group, and they all increase in the bone tissue of osteoporosis mice. We therefore concluded that osteoporosis indeed induces fatty acyl metabolic disorders.

In addition to fatty acyls, glycerolipids (primarily TG), sterols, some glycerophospholipids, and ceramides (Cer) also increased in the PMOP mice. Conversely, another part of glycerophosphocholines (PC) and sphingolipids showed a downward trend. Triglycerides belong to the glycerolipids group. Qi *et al*. reported that TG increased in postmenopausal women obviously^18^, and Xue *et al*. also found that TG significantly increased in PMOP mice^42^. Additional studies reported that lipid metabolism is regulated by peroxisome proliferator activated receptor γ (PPARγ), and the accumulation of lipids increases its oxidation, resulting in the activation of PPARγ. The activation of PPARγ could not only inhibit the differentiation of osteoblasts but also promote bone marrow stromal cell differentiation into adipogenic cells^43,44^. Lysophosphatidylcholines (LPC) and phosphatidylcholines (PC) belong to the glycerophosphate group. These phospholipids are an integral component of the animal cell membrane structure and can mutually transform one into another. The increase of LPC and PC is suggestive of an oxidative stress response. LPC is reportedly significantly up-regulated in the plasma of osteoporosis mice, and increased ROS leads to oxidative stress damage^45^. The existence of oxidative stress may lead to increased bone mass loss and bone friability, thereby aggravating the process of osteoporosis^46^. In our study, TG together with glycerophospholipids (e.g., PC, PA, PE, and PS) were found to be disordered in osteoporosis mice, suggesting that the abnormal metabolism of glycerolipids and glycerophospholipids is related to osteoporosis.

Sphingolipids, including sphingomyelin (SM), glycosphingolipid and ceramide, were also found to be altered in our study. Among these sphingolipids, sphingomyelins regulate cell growth and differentiation and function as second messengers^47^. Glycosphingolipids, components of the cell membrane, play an important role in tissue development and function and can regulate trans membrane signals and mediate the interaction between cells and cells or cells and the matrix^48^. Glycosphingolipids are considered to be essential for the formation of osteoblasts induced by RANKL^49^. Increased glycosphingolipids can accelerate the differentiation and formation of osteoblasts, destroying the original balance in the bone tissue. Under the continuous action of acidic hydrolase, glycosphingolipid can be degraded into ceramide, which is an inhibitor of protein transport, secretion, and inhibit proliferation and promote apoptosis^50^. In our study, sphingomyelins are obviously decreased in the bone tissue of PMOP mice, while glycosphingolipids and ceramides are increased, suggesting the dysregulated sphingolipid metabolism is inseparable from osteoporosis. In addition, increased steroids are able to promote bone resorption, especially vitamin D3 and its products and sterol ester analogues, which can induce the formation of osteoclasts and destroy bone tissue, resulting in osteoporosis^51^.

This study has several limitations. First, the sample size used in the metabolomics and lipidomics analyses only meet the statistical power requirements. Additional studies with larger sample sizes will bolster our initial results. Secondly, we did not perform a systematic study on metabolites in vertebrae, which are another representative sample of osteoporosis; however, we believe that further experiments are warranted to elucidate the differentially expressed metabolites in vertebrae to more completely explain the mechanisms and metabolism disorders associated with PMOP.

## Materials and Methods

### Chemicals and reagents

Acetonitrile (ACN) (LC–MS optimum grade) and methanol (HPLC grade) were purchased from Merck (Darmstadt, Germany); Formic acid was obtained from Fluka (Buchs, Switzerland). Deionized water from a Millipore Milli-Q water purification system (Millipore Corp., Billerica, MA, USA) were used to prepare the chromatographic mobile phases. The reference standards of L-Leucine, L-Phenylalanine, L-Tryptophan, L-Arginine, Xanthine, and Hypoxanthine were supplied by Sigma Corporation (St. Louis, MO, USA). All other chemicals were of analytical grade.

### OVX model construction

Eighteen wild-type female C57B/L6 mice aged 8-week-old were purchased from Shanghai SLAC laboratory Animal Co. Ltd. After conditional housing for one week under controlled temperature (22–24 °C), humidity(50–60%), a 12 h light/dark cycle and free water and food. Mice were randomly divided into sham group (Sham) and ovariectomy group (OVX). The ovariectomized mouse model were performed in the specific pathogen free animal laboratoryas described previously^52^ in OVX group to induce osteoporosis under chloral hydrate anesthesia, and the rest mice receive a sham procedure. After being housed for 8 weeks, all animals’ femur samples were collected by removing tissue. 3 of each group were randomly selected and fixed in 4% paraformaldehyde 48 h for Micro CT and histological study, the rest were quickly placed in liquid nitrogen and stored at −80 °C after the bone marrow was completely flushed out. All animal studies were performed in accordance with the National Institutes of Health (NIH) guide for the Care and Use of Laboratory Animals and approved by the Ethical Committee for the Experimental Use of Animals at Second Military Medical University (Shanghai, China).

### Histomorphometric analysis using micro-CT and HE staining

We analyzed 100 section planes from the growth plate in each femurs bone using a high-performance micro-CT (Skyscan1172, Antwerp, Belgium) and detected the metaphyseal region and trabecular bone by built-in software to obtain the following parameters within the region of interest: Bone mineral density (BMD), bone volume/total volume (BV/TV), bone surface area/total volume (BS/TV), Trabecular number (Tb, N). Femur samples were decalcified in 10% EDTA with continuous shaking for 3 weeks. Each distal femur was sliced for 4 μm sections and performed for H&E staining.

### Metabolic and lipidomic analysis of femur samples

#### Sample preparation

Bone tissue was cleaned of soft tissue, bone marrow, cartilage, and periosteum, then flash frozen in liquid nitrogen. Every femur sample was crumbed and grounded into powder in liquid nitrogen. For metabolic analysis, 300 μl pre-cooling 80% methanol solution which contained 4 µg/ml 2-Chloro-L-phenylalanine as internal standard was added in each weighted sample (Weighing about 20 ± 2 mg, accurately) for protein precipitation and homogenate using a high-throughput tissue grinder (teice, 50 Hz, 2 min). Subsequently, the mixtures were centrifuged at 13,000rmp, 4 °C for 15 min, aliquots (10 µL) of each supernatant were mixed to generate a pooled quality control (QC) sample. For lipidomic analysis, the mice femurs powder were weighed(about 20 mg) and added with 190 ul methanol-chloroform-water (2:1:0.8, v/v/v), vortex for 1 minute and sonication for 10 minutes in 4 °C to extract the lipids. Then, 1 volume of chloroform and 1 volume of water were added, vortexed for 5 min and centrifuged at 13000 rpm with 4 °C for 10 minutes, and then 50 μl chloroform layer was dried with nitrogen. Finally, the residue was reconstituted with 100 μl isopropanol-acetonitrile-water (2:1:1, v/v/v), and the supernatant was prepared to be tested with 10 µL of each supernatant were mixed to generate a pooled quality control (QC) sample.

#### UPLC–MS/MS conditions

The UPLC–MS/MS analysis was carried out using an Agilent 1290 Infinity system together with an Agilent 6538 UHD Quadrupole Time-of-Flight mass spectrometer (Agilent, USA). For metabolic analysis, chromatographic separations were performed on an X Bridge® BEH Amide column (2.1 mm*100 mm* 2.5 µm, Waters) at a column oven temperature of 30 °C. The initial mobile phase was a mixture of 98% phase A (0.1% formic acid in water) and 2% phase B (0.1% formic acid in ACN). After injection, the mobile phase was according to the following gradient eluted condition: 0–2 min, 95% B; 2–4 min, 95–89% B; 4–13.5 min, 89% B; 13.5–15 min, 89–66% B; 15–17 min, 66% B then 7 min were required to re-establish the initial conditions. For lipidomic analysis, chromatographic separations were performed at 45 °C on a Waters X Bridge^TM^ BEH C18 analytical column (2.1 mm × 100 mm, 2.5μm, Waters, Milford, MA). The mobile phase consisted of 40:60 water: ACN with 0.1% formic acid and 10 mM Ammonium formate (C) and 9:10:81 ACN: water: isopropanol with 0.1% formic acid and 10 mM Ammonium formate (D). The detailed gradient elution conditions were as follow: 0–3 min, 40–68%D; 3–5 min, 68–70%D; 5–7 min, 70%D; 7–12 min, 70–85%D; 12–15 min, 85–99%D; 15–19 min, 99%D; 19–19.5 min, 99–40%D; 19.5–20 min, 40%D. The flow rate was set to 0.4 ml/min and the auto-sampler was maintained at 4 °C. The injected volume was 3 μL and the samples were randomly run. QC samples were analyzed after every 4 injections to ascertain that the mass spectrometer performance was stable during the analysis of the samples set.

An electrospray ionization source (ESI) was used in both positive and negative ion mode with the ion spray voltage was set to 4000 V and −3500 V respectively. Other parameters were as follows: drying gas flow, 11 L/min; gas temperature, 350 °C; Nebulizer pressure, 45 psig; fragmentor voltage, 120 V; skimmer voltage, 60 V. Octopole RF Peak, 750 V; reference masses (m/z), 121.0509 Da and 922.0098 Da. The MS data were collected in the full scan mode ranging from 50 to 1100 m/z. The biomarker candidates were further analyzed by MS/MS with the collision energy 10, 20 and 40 eV.

### Data analysis and statistical analysis

After the UPLC/Q-TOF-MS analysis, the raw data (·d) were converted into a common data format (.mz data) files using Agilent Mass Hunter workstation software version B.01.04 (Agilent, MA, USA) with the isotope interferences being excluded and the intensity threshold was set to 300 to exclude the noise. Then, the data from each polarity were processed using the open-free XCMS^53^ (http://metlin.scripps.edu/download/) for peak extraction, alignment and integration to generate a visual data matrix. There are 1232 and 370 features in positive and negative mode in metabolomics, while in lipidomics there are 3168 and 267 features in positive and negative mode. Then only molecular entities detected in at least 80% of the samples belonging to at least one of the groups under study (OVX and Sham individuals) were considered. After filtering, there are 633 and 162 features in positive and negative mode in metabolomics, while in lipidomics there are 1571 and 67 features in positive and negative mode. Normalization of all detected ions in each sample were carried out by the peak area of internal standard in each sample to obtain the relative intensity then further normalized by the weight of each sample in metabolomics analysis; while in lipidomics analysis, Normalization procedure was performed by the weight of each sample and then analyzed by peak area normalizing method. Finally, three three-dimensional data matrix, including the sample names, retention times, m/z pairs, and normalized ion intensities, were imported into the SIMCA-P program (version 11.0, Umetrics, Umea, Sweden) for multivariate statistical analysis including principal component analysis (PCA) and partial least squares-discriminant analysis (PLS-DA). The relevant parameters R^2^X, R^2^Y, and Q^2^ of PLS-DA model were monitored to evaluate the goodness of fit and prediction internally and permutation tests were implemented to evaluate the quality of models externally. VIP (variable importance) value, generated in PLS-DA processing, represents the contribution to the group discrimination of each metabolite^54^. In our study, VIP values of all potential biomarkers should be greater than 1.

All data were expressed by means ± SD. The statistical significance in of mean values was tested using students T-test through SPSS 17.0 program (IBM, New York, USA). Differences were considered to be significant when p values were less than 0.05.

### Identification of potential biomarkers

Potential biomarkers were identified as the previous report^54^. The ions were confirmed based on the extracted ion chromatogram (EIC) and then compared MS and MS/MS information by searching in the METLIN MS and MS/MS database (http://metlin.scripps.edu) and the Human Metabolome Database (http://www.hmdb.ca/). An accuracy error of 10 ppm was set in MS searching and the fragments were verified by MS/MS data; furthermore, the retention times and the fragments of metabolites were compared with those of reference samples to confirm the identification of metabolites. Besides, a part of lipids were tentatively identified based on their characteristic molecular ion information and corresponding fragments of product ion. We take Supplementary Figs S4 and S5 which was validated based on metlin database and their fragment ions to illustrate the identification process.

## Conclusions

In this study, bilateral ovariectomy was performed to establish a mouse model of osteoporosis, and UPLC/Q-TOF-MS based lipidomics in combination with polar metabolomics were used to build a comprehensive metabolic profile of the mouse femur, showing an overall change from lipid metabolites to polar metabolites. We found there were 11 polar metabolites and 93 lipid metabolites that were significantly changed. These metabolites are involved in amino acid metabolism, nucleotide metabolism and lipid metabolism. Among the lipids, fatty acyls, glycerolipids, glycerophospholipids, sphingolipids and sterols were altered. These dysregulated metabolic pathways, especially lipid metabolism, revealed a series of metabolic disorders caused by changes in the hormone levels after ovariectomy and are related to the imbalance between bone resorption and formation. The lipidomics in combination with polar metabolomics methods used in this study show good applicability and wide coverage in the metabolic profile of bone tissue, especially for the lipidomics profile, and fill the gap in the field of bone tissue metabolite analysis. Furthermore, the metabolite information of the OVX mouse bone tissue obtained in this study provides a foundation for understanding the differentially regulated pathways and mechanisms of PMOP.

## Electronic supplementary material

Supplementary File
