# Supplementary material for: Integrative Bone Metabolomics—Lipidomics Strategy for Pathological Mechanism of Postmenopausal Osteoporosis Mouse Model
Source: Sci Rep. 2018 Nov 7;8:16456. doi: 10.1038/s41598-018-34574-6 (PMC6220250; doi:10.1038/s41598-018-34574-6)
Supplement: Supplementary file 1 — Supplementary File [file 41598_2018_34574_MOESM1_ESM.docx]

**Integrative Bone Metabolomics−Lipidomics Strategy for Pathological Mechanism of Postmenopausal Osteoporosis Mouse Model**

Hongxia Zhao^a,e,1^, Xiaoqun Li^b,1^, Dianying Zhang ^c,1^, Haiyan Chen^d^ , Yufan Chao^a^, Kaiwen Wu^e^, Xin Dong^a*^, Jiacan Su^f**^

**Additional Information：There is no competing interest and non-financial competing interests.**^a^ School of Pharmacy, Second Military Medical University, Shanghai 200433, China.

^b^ Graduate Management Unit, Shanghai Changhai Hospital, Second Military Medical University, Shanghai 200433, China

^c^ Department of Orthopedics Trauma, Peking University People's Hospital, Beijing 100009, China.

^d^ Zhongshan Hospital of Fudan University, 180 Fenglin Road, Shanghai 200032, China

^e^ Nine Company, Faculty of Medicine, Second Military Medical University, Shanghai 200120, China.

^f^ Department of Orthopedics Trauma, Shanghai Changhai Hospital, Second Military Medical University, Shanghai 200433, China.

^1^ These authors contributed equally to this work.

Corresponding author: *****E-mail: [dongxinsmmu@126.com](mailto:dongxinsmmu@126.com); ******E-mail:[drsujiacan@163.com](mailto:drsujiacan@163.com), Fax: +86 21- 81871335; Tel/Fax: +86 21- 31161699

**Table S1 Differential lipids expressed in OVX group**

| **No.** | **Name** | **Formula** | **m/z** | **Adduct** | **Rt**  **(min)** | **FC**  **(M-C)** | **P(M-C)** | **FDR** | | **Category** |
| --- | --- | --- | --- | --- | --- | --- | --- | --- | --- | --- |
| 1 | cis-5-dodecenoic acid ^a^ | C_12_H_22_O_2_ | 199.1690 | [M+H]^+^ | 1.96 | 3.27↑ | 3.09E-07*** | | 1.8226E-04 | Fatty Acyls-Fatty acid |
| 2 | 5Z-undecenoic acid ^b^ | C_11_H_20_O_2_ | 202.1804 | [M+NH4]^+^ | 0.82 | 3.19↑ | 2.54E-06*** | | 3.2220E-03 | Fatty Acyls-Fatty acid |
| 3 | 10-hydroxy-16-oxo-hexadecanoic acid ^c^ | C_16_H_30_O_4_ | 287.2218 | [M+H]^+^ | 1.96 | 3.26↑ | 3.13E-07*** | | 2.5949E-04 | Fatty Acyls-Fatty acid |
| 4 | Corchorifatty acid A ^b^ | C_18_H_28_O_4_ | 309.2035 | [M+H]^+^ | 1.96 | 3.66↑ | 4.22E-07*** | | 6.1178E-04 | Fatty Acyls-Fatty acid |
| 5 | 7,8-epoxy-17S-HDHA ^b^ | C_22_H_30_O_4_ | 376.2476 | [M+NH4]^+^ | 1.07 | 3.54↑ | 5.32E-07*** | | 1.0269E-04 | Fatty Acyls-Fatty acid |
| 6 | 4Z,7Z,10Z,13Z-hexadecatetraenoic acid ^b^ | C_16_H_24_O_2_ | 293.1761 | [M+FA-H]^-^ | 0.96 | 4.89↑ | 3.42E-07*** | | 1.2052E-06 | Fatty acyls-Fatty acid |
| 7 | 1-Hexadecylamine ^c^ | C_16_H_35_N | 242.2842 | [M+H]^+^ | 2.24 | 2.80↑ | 2.67E-06*** | | 2.4505E-03 | Fatty Acyls-Fatty amides |
| 8 | N-dodecyl-Octadecanamide ^c^ | C_30_H_61_NO | 452.4822 | [M+H]^+^ | 9.41 | 3.36↑ | 1.67E-06*** | | 1.6424E-03 | Fatty Acyls-Fatty amides |
| 9 | Capsi-amide ^c^ | C_17_H_35_NO | 270.2790 | [M+H]^+^ | 3.61 | 3.52↑ | 3.35E-07*** | | 4.7976E-04 | Fatty Acyls-Fatty amides |
| 10 | 1-Octadecanamine ^a^ | C_18_H_36_ | 270.3153 | [M+H]^+^ | 2.98 | 2.93↑ | 2.86E-06*** | | 1.8728E-03 | Fatty Acyls-Fatty amides |
| 11 | Dimyristylamine ^b^ | C_28_H_59_N | 410.4717 | [M+H]^+^ | 4.48 | 3.14↑ | 9.19E-07*** | | 9.7632E-04 | Fatty Acyls-Fatty amides |
| 12 | Stearamide ^c^ | C_18_H_37_NO | 284.2946 | [M+H]^+^ | 3.85 | 3.68↑ | 7.48E-07*** | | 8.7774E-04 | Fatty Acyls-Fatty amides |
| 13 | Palmitoyl N-Isopropylamide ^b^ | C_19_H_39_NO | 298.3104 | [M+H]^+^ | 4.42 | 3.54↑ | 5.82E-07*** | | 5.2232E-04 | Fatty Acyls-Fatty amides |
| 14 | Oleoyl Ethyl Amide ^c^ | C_20_H_39_NO | 310.3103 | [M+H]^+^ | 3.89 | 6.26↑ | 1.30E-05*** | | 1.2514E-02 | Fatty Acyls-Fatty amides |
| 15 | 13Z-Docosenamide ^a^ | C_22_H_43_NO | 338.3415 | [M+H]^+^ | 4.66 | 5.76↑ | 6.95E-06*** | | 1.5809E-03 | Fatty Acyls-Fatty amides |
| 16 | N-butyl arachidonoyl amine ^b^ | C_2_4H_41_NO | 360.3234 | [M+H]^+^ | 4.66 | 3.67↑ | 1.68E-06*** | | 8.8283E-04 | Fatty Acyls-Fatty amides |
| 17 | N-Hexadecylhexadecanamide ^b^ | C_32_H_65_NO | 480.5133 | [M+H]^+^ | 11.03 | 3.11↑ | 5.45E-07*** | | 2.7083E-04 | Fatty Acyls-Fatty amides |
| 18 | N-octadecylhexadecanamide ^b^ | C_34_H_69_NO | 508.5446 | [M+H]^+^ | 12.55 | 2.67↑ | 2.44E-05*** | | 9.4983E-03 | Fatty Acyls-Fatty amides |
| 19 | N,N'-1,2-ethanediylbis-hexadecanamide ^b^ | C_34_H_68_N_2_O_2_ | 537.5346 | [M+H]^+^ | 9.48 | 4.98↑ | 1.31E-06*** | | 1.8176E-03 | Fatty Acyls-Fatty amides |
| 20 | N-Docosyldocosanamide ^b^ | C_44_H_89_NO | 648.7008 | [M+H]^+^ | 16.53 | 6.74↑ | 3.01E-06*** | | 4.5561E-03 | Fatty Acyls-Fatty amides |
| 21 | 10E,12E-Hexadecadien-1-ol^a^ | C_16_H_30_O | 256.2633 | [M+NH4]^+^ | 4.05 | 3.33↑ | 1.52E-06*** | | 2.1177E-03 | Fatty Acyls-Fatty alcohols |
| 22 | (+)-1,18-Nonacosanediol ^b^ | C_29_H_60_O_2_ | 463.4478 | [M+Na]^+^ | 11.76 | 5.23↑ | 5.05E-07*** | | 4.6618E-04 | Fatty Acyls-Fatty alcohols |
| 23 | 1,7-Pentatriacontadien-11-ol^c^ | C_35_H_68_O | 522.5604 | [M+NH4]^+^ | 14.89 | 4.98↑ | 4.29E-07*** | | 3.9377E-04 | Fatty Acyls-Fatty alcohols |
| 24 | (6R*,8S*)-6,8-Tetratriacontanediol ^b^ | C_34_H_70_O_2_ | 533.5261 | [M+Na]^+^ | 15.17 | 6.68↑ | 8.59E-07*** | | 3.3104E-04 | Fatty Acyls-Fatty alcohols |
| 25 | 11Z-Eicosenal^c^ | C_20_H_38_O | 312.3259 | [M+H]^+^ | 4.05 | 3.27↑ | 1.49E-06*** | | 2.1974E-03 | Fatty Acyls-Fatty aldehydes |
| 26 | Myristic Acid ethyl ester ^b^ | C_16_H_32_O_2_ | 255.2328 | [M-H]- | 2.33 | 2.38↑ | 1.34E-06*** | | 1.9524E-06 | Fatty Acyls -fatty esters |
| 27 | Palmitic Acid ethyl ester ^b^ | C_18_H_36_O_2_ | 283.2642 | [M-H]- | 3.62 | 2.01↑ | 4.41E-05*** | | 6.1493E-05 | Fatty Acyls -fatty esters |
| 28 | Arachidyl linoleate ^b^ | C_38_H_72_O_2_ | 561.5574 | [M+H]^+^ | 16.09 | 6.83↑ | 8.54E-07*** | | 7.0566E-04 | Fatty Acyls-Fatty esters |
| 29 | 13,14-dihydro-15-keto-tetranor PGE2 ^b^ | C_16_H_26_O_5_ | 316.2123 | [M+NH4]^+^ | 0.83 | 3.81↑ | 4.79E-07*** | | 3.3714E-04 | Fatty Acyls-Eicosanoids |
| 30 | 3-Hydroxy-11Z-octadecenoylcarnitine ^b^ | C_25_H_47_NO_5_ | 442.3537 | [M+H]^+^ | 5.33 | 3.44↑ | 3.67E-07*** | | 5.2533E-04 | Fatty Acyls -fatty esters |
| 31 | 3-Hydroxy-cis-5-tetradecenoylcarnitine ^b^ | C_21_H_39_NO_5_ | 420.2510 | [M+Cl]^-^ | 0.96 | 4.36↑ | 9.48E-07*** | | 4.2334E-06 | Fatty Acyls -fatty esters |
| 32 | PG(P-16:0/14:1) ^b^ | C_24_H_52_NO_6_P | 482.3604 | [M+H]^+^ | 2.69 | 0.68↓ | 9.99E-04*** | | 5.7179E-04 | Glycerophospholipids |
| 33 | PA(P-16:0/16:1) ^b^ | C_36_H_69_O_9_P | 694.4951 | [M+NH4]^+^ | 8.57 | 4.98↑ | 3.92E-07*** | | 2.3974E-05 | Glycerophospholipids-Glycerophosphoglycerols |
| 34 | PA(P-16:0/16:0) ^b^ | C_35_H_67_O_7_P | 631.4694 | [M+H]^+^ | 10.39 | 2.86↑ | 1.88E-08*** | | 8.6278E-05 | Glycerophospholipids-Glycerophosphates |
| 35 | PA(14:1/18:0) ^b^ | C_35_H_69_O_7_P | 633.4849 | [M+H]^+^ | 11.73 | 2.88↑ | 6.33E-08*** | | 1.5358E-04 | Glycerophospholipids-Glycerophosphates |
| 36 | PA(P-16:0/18:2) ^b^ | C_35_H_67_O_8_P | 647.4583 | [M+H]^+^ | 12.72 | 4.43↑ | 6.21E-07*** | | 5.1210E-05 | Glycerophospholipids-Glycerophosphates |
| 37 | PA(P-16:0/18:1) ^b^ | C_37_H_69_O_7_P | 657.4852 | [M+H]^+^ | 10.58 | 3.89↑ | 5.48E-08*** | | 7.4564E-05 | Glycerophospholipids-Glycerophosphates |
| 38 | PA(13:0/22:6) ^b^ | C_37_H_71_O_7_P | 659.5007 | [M+H]^+^ | 11.92 | 3.50↑ | 6.63E-08*** | | 1.6777E-03 | Glycerophospholipids-Glycerophosphates |
| 39 | PA(22:0/22:2) ^b^ | C_38_H_63_O_8_P | 701.4092 | [M+Na]^+^ | 8.64 | 5.45↑ | 1.93E-06*** | | 5.8344E-04 | Glycerophospholipids-Glycerophosphates |
| 40 | PA(22:0/22:0) ^b^ | C_47_H_89_O_8_P | 813.6361 | [M+H]^+^ | 15.94 | 5.76↑ | 4.41E-07*** | | 2.4708E-04 | Glycerophospholipids-Glycerophosphates |
| 41 | PC(O-16:0/16:0)^c^ | C_47_H_93_O8P | 839.6518 | [M+Na]^+^ | 15.99 | 7.24↑ | 3.06E-07*** | | 5.6241E-02 | Glycerophospholipids-Glycerophosphates |
| 42 | PC(16:0/16:1)^c^ | C_40_H_78_NO_8_P | 732.5538 | [M+H]^+^ | 7.29 | 1.90↑ | 3.16E-06*** | | 2.2588E-03 | Glycerophospholipids-Glycerophosphocholines |
| 43 | 1,2-dipalmitoyl-sn-glycero-3-PC^c^ | C_40_H_80_NO_8_P | 734.5692 | [M+H]^+^ | 8.93 | 1.37↑ | 2.91E-04*** | | 4.2256E-05 | Glycerophospholipids-Glycerophosphocholines |
|  | 1,2-dipalmitoyl-sn-glycero-3-PC^c^ | C_40_H_80_NO_8_P | 756.5512 | [M+Na]^+^ |  |  |  |  |  |  |
| 44 | PC(O-16:0/18:2)^c^ | C_42_H_82_NO_7_P | 744.5899 | [M+H]^+^ | 8.74 | 0.53↓ | 9.23E-08*** | | 6.3579E-05 | Glycerophospholipids-Glycerophosphocholines |
| 45 | PC(16:0/18:1)^c^ | C_42_H_82_NO_8_P | 760.5848 | [M+H]^+^ | 9.02 | 1.48↑ | 1.25E-04*** | | 5.0998E-02 | Glycerophospholipids-Glycerophosphocholines |
| 46 | Arachidonoyl PAF C-16^c^ | C_44_H_82_NO_7_P | 768.5899 | [M+H]^+^ | 8.41 | 0.66↓ | 9.85E-06*** | | 7.1107E-03 | Glycerophospholipids-Glycerophosphocholines |
| 47 | PC(O-18:1(9Z)/18:2)^c^ | C_44_H_84_NO_7_P | 770.6056 | [M+H]^+^ | 8.77 | 0.63↓ | 1.96E-06*** | | 7.1107E-03 | Glycerophospholipids-Glycerophosphocholines |
| 48 | PC(O-18:0/18:2)^c^ | C_44_H_86_NO_7_P | 772.6209 | [M+H]^+^ | 10.30 | 0.52↓ | 4.02E-06*** | | 4.8898E-03 | Glycerophospholipids-Glycerophosphocholines |
| 49 | PC(16:0/20:4)^c^ | C_44_H_80_NO_8_P | 782.5693 | [M+H]^+^ | 7.35 | 1.69↑ | 5.21E-05*** | | 2.3382E-02 | Glycerophospholipids-Glycerophosphocholines |
| 50 | PC(O-16:0/22:6)^c^ | C_46_H_82_NO_7_P | 792.5898 | [M+H]^+^ | 7.86 | 0.59↓ | 4.15E-07*** | | 4.6405E-04 | Glycerophospholipids-Glycerophosphocholines |
| 51 | PC(16:0/20:4)^c^ | C_44_H_80_NO_8_P | 804.5511 | [M+Na]^+^ | 7.37 | 2.81↑ | 1.36E-07*** | | 1.4739E-04 | Glycerophospholipids-Glycerophosphocholines |
| 52 | PC(24:0/22:6) ^b^ | C_54_H_96_NO_8_P | 918.7018 | [M+H]^+^ | 16.28 | 3.59↑ | 2.74E-06*** | | 3.8944E-03 | Glycerophospholipids-Glycerophosphocholines |
| 53 | PC(24:0/22:5) ^b^ | C_54_H_98_NO_8_P | 920.7174 | [M+H]^+^ | 16.85 | 4.52↑ | 2.11E-04*** | | 2.7676E-01 | Glycerophospholipids-Glycerophosphocholines |
| 54 | PE(P-18:1/22:6) ^b^ | C_45_H_76_NO_7_P | 796.5251 | [M+Na]^+^ | 7.61 | 2.12↑ | 7.99E-06*** | | 9.3603E-03 | Glycerophospholipids-Glycerophosphoethanolamines |
| 55 | PS(18:0/22:0) ^b^ | C_46_H_90_NO_10_P | 865.6677 | [M+NH4]^+^ | 16.07 | 5.15↑ | 2.65E-07*** | | 2.1417E-04 | Glycerophospholipids-Glycerophosphoserines |
| 56 | PS(P-16:0/14:1) ^b^ | C_36_H_68_NO_9_P | 707.4907 | [M+NH4]^+^ | 8.56 | 5.03↑ | 2.73E-07*** | | 1.7738E-04 | Glycerophospholipids-Glycerophosphoserines |
| 57 | PS(20:1/22:0) ^b^ | C_48_H_92_NO_10_P | 891.6834 | [M+NH4]^+^ | 16.16 | 4.45↑ | 6.07E-07*** | | 5.6193E-04 | Glycerophospholipids-Glycerophosphoserines |
| 58 | PS(22:1/22:1) ^b^ | C_50_H_94_NO_10_P | 917.6984 | [M+NH4]^+^ | 16.28 | 3.61↑ | 2.35E-06*** | | 3.3080E-03 | Glycerophospholipids-Glycerophosphoserines |
| 59 | PS(22:0/22:1) ^b^ | C_50_H_96_NO_10_P | 919.7149 | [M+NH4]^+^ | 16.80 | 3.61↑ | 1.09E-05*** | | 9.0470E-03 | Glycerophospholipids-Glycerophosphoserines |
| 60 | PS(22:0/22:0) ^b^ | C_50_H_98_NO_10_P | 921.7305 | [M+NH4]^+^ | 17.38 | 4.27↑ | 7.20E-07*** | | 5.0979E-04 | Glycerophospholipids-Glycerophosphoserines |
| 61 | TG(14:0/16:1/16:1)^c^ | C_49_H_90_O_6_ | 792.7067 | [M+NH4]^+^ | 15.95 | 8.05↑ | 9.32E-06*** | | 1.1658E-02 | Glycerolipids-Triacylglycerols |
| 62 | TG(14:0/16:1/16:0)^c^ | C_49_H_92_O_6_ | 794.7225 | [M+NH4]^+^ | 16.55 | 5.83↑ | 8.42E-06*** | | 1.0091E-02 | Glycerolipids-Triacylglycerols |
| 63 | TG(16:1/14:0/18:2)^c^ | C_51_H_92_O_6_ | 818.7226 | [M+NH4]^+^ | 16.00 | 10.70↑ | 1.43E-05*** | | 1.0521E-02 | Glycerolipids-Triacylglycerols |
| 64 | TG(16:0/16:1/16:1)^c^ | C_51_H_94_O_6_ | 820.7389 | [M+NH4]^+^ | 16.58 | 7.72↑ | 1.26E-05*** | | 6.3359E-03 | Glycerolipids-Triacylglycerols |
| 65 | TG(14:0/18:1/16:0)^c^ | C_51_H_96_O_6_ | 822.7542 | [M+NH4]^+^ | 17.24 | 5.07↑ | 1.23E-05*** | | 8.5465E-03 | Glycerolipids-Triacylglycerols |
| 66 | TG(16:0/16:1/16:1)^c^ | C_51_H_94_O_6_ | 825.6935 | [M+Na]^+^ | 16.57 | 5.84↑ | 7.95E-07*** | | 1.1995E-03 | Glycerolipids-Triacylglycerols |
| 67 | TG(16:1/16:0/18:2)^c^ | C_53_H_96_O_6_ | 846.7544 | [M+NH4]^+^ | 16.66 | 7.93↑ | 2.22E-05*** | | 7.5632E-03 | Glycerolipids-Triacylglycerols |
| 68 | TG(16:0/16:1/18:1)^c^ | C_53_H_98_O_6_ | 848.7700 | [M+NH4]^+^ | 17.26 | 4.88↑ | 3.31E-05*** | | 1.2427E-02 | Glycerolipids-Triacylglycerols |
| 69 | TG(16:0/16:0/18:1)^c^ | C_53_H_100_O_6_ | 850.7856 | [M+NH4]^+^ | 18.03 | 3.38↑ | 7.10E-05*** | | 5.5380E-02 | Glycerolipids-Triacylglycerols |
| 70 | TG(16:0/18:2/16:1)^c^ | C_53_H_96_O_6_ | 851.7094 | [M+Na]^+^ | 16.66 | 6.39↑ | 5.08E-07*** | | 5.1527E-04 | Glycerolipids-Triacylglycerols |
| 71 | TG(16:0/16:1/18:1) ^b^ | C_53_H_98_O_6_ | 853.7248 | [M+Na]^+^ | 17.25 | 4.17↑ | 8.89E-07*** | | 1.1004E-03 | Glycerolipids-Triacylglycerols |
| 72 | TG(16:0/18:2/18:1)^c^ | C_55_H_100_O_6_ | 874.7855 | [M+NH4]^+^ | 17.36 | 5.60↑ | 4.28E-05*** | | 1.2740E-02 | Glycerolipids-Triacylglycerols |
| 73 | TG(16:0/18:1/18:1)^c^ | C_55_H_102_O_6_ | 876.8013 | [M+NH4]^+^ | 18.04 | 4.43↑ | 6.98E-05*** | | 3.6071E-02 | Glycerolipids-Triacylglycerols |
| 74 | TG(16:1/18:1/18:2)^c^ | C_55_H_98_O_6_ | 877.7251 | [M+Na]^+^ | 16.71 | 4.43↑ | 4.52E-06*** | | 4.7292E-03 | Glycerolipids-Triacylglycerols |
| 75 | TG(18:1/16:1/18:1)^c^ | C_55_H_100_O_6_ | 879.7407 | [M+Na]^+^ | 17.36 | 4.87↑ | 6.08E-07*** | | 5.7552E-04 | Glycerolipids-Triacylglycerols |
| 76 | TG(18:1/16:0/18:1) ^b^ | C_55_H_102_O_6_ | 881.7560 | [M+Na]^+^ | 18.04 | 4.21↑ | 1.96E-06*** | | 2.7696E-03 | Glycerolipids-Triacylglycerols |
| 77 | TG(18:1/18:2/18:2)^c^ | C_57_H_100_O_6_ | 898.7856 | [M+NH4]^+^ | 16.79 | 4.04↑ | 1.42E-03** | | 2.7192E-02 | Glycerolipids-Triacylglycerols |
|  | TG(18:1/18:2/18:2) ^b^ | C_57_H_100_O_6_ | 903.7405 | [M+Na]^+^ |  |  |  |  |  |  |
| 78 | TG(18:1/18:1/18:2)^c^ | C_57_H_102_O_6_ | 900.8013 | [M+NH4]^+^ | 17.38 | 4.47↑ | 4.78E-04*** | | 7.0151E-03 | Glycerolipids-Triacylglycerols |
|  | TG(18:1/18:1/18:2) ^b^ | C_57_H_102_O_6_ | 905.7562 | [M+Na]^+^ |  |  |  |  |  |  |
| 79 | TG(18:1/18:1/18:1)^c^ | C_57_H_104_O_6_ | 902.8169 | [M+NH4]^+^ | 18.05 | 4.93↑ | 6.69E-05*** | | 4.1150E-02 | Glycerolipids-Triacylglycerols |
| 80 | SM(d18:1/16:0)^c^ | C_39_H_79_N_2_O_6_P | 725.5564 | [M+Na]^+^ | 7.26 | 3.05↑ | 5.37E-08*** | | 6.1856E-05 | Sphingolipids-sphingomyelins |
| 81 | SM(d18:1/18:0) ^b^ | C_41_H_83_N_2_O_6_P | 731.6059 | [M+H]^+^ | 9.10 | 1.55↑ | 6.02E-05*** | | 8.8287E-02 | Sphingolipids-sphingomyelins |
| 82 | SM(d18:2/24:1) ^b^ | C_47_H_91_N_2_O_6_P | 811.6685 | [M+H]^+^ | 10.86 | 0.66↓ | 1.23E-05*** | | 1.1067E-02 | Sphingolipids-sphingomyelins |
| 83 | SM(d18:1/24:0) ^b^ | C_47_H_95_N_2_O_6_P | 815.6993 | [M+H]^+^ | 12.65 | 0.62↓ | 1.02E-06*** | | 1.5709E-03 | Sphingolipids-sphingomyelins |
| 84 | SM(d18:0/16:0) ^b^ | C_39_H_81_N_2_O_6_P | 705.5901 | [M+H]^+^ | 7.93 | 0.65↓ | 5.02E-06*** | | 7.3689E-03 | Sphingolipids-sphingomyelins |
| 85 | Galα1-3(Fucα1-2)Galβ1-4Glcβ-Cer(d18:1/26:1) ^b^ | C_68_H_125_NO_22_ | 1325.8996 | [M+NH4]^+^ | 8.63 | 3.97↑ | 3.92E-07*** | | 1.1513E-04 | Sphingolipids-glycosphingolipids |
| 86 | Cer(d14:2/20:1) ^b^ | C_34_H_63_NO_3_ | 551.5138 | [M+NH4]^+^ | 6.36 | 3.70↑ | 1.52E-06*** | | 2.3681E-03 | Sphingolipids-Ceramides |
| 87 | Ceramide (d18:1/16:0) ^b^ | C_34_H_67_NO_3_ | 582.5108 | [M+FA-H]^-^ | 7.86 | 1.72↑ | 3.22E-07*** | | 3.9998E-07 | Sphingolipids-Ceramides |
| 88 | Cer(d18:1/24:1(15Z)) ^b^ | C_42_H_81_NO_3_ | 692.6204 | [M+FA-H]^-^ | 13.07 | 1.40↑ | 4.94E-05*** | | 5.4286E-05 | Sphingolipids-Ceramides |
| 89 | PI-Cer(d18:0/18:0(2OH)) ^b^ | C_42_H_84_NO_12_P | 848.5561 | [M+Na]^+^ | 9.15 | 3.24↑ | 3.53E-08*** | | 4.5970E-05 | Sphingolipids-Ceramide phosphoinositols |
| 90 | PI-Cer(d18:0/16:0(2OH)) ^b^ | C_40_H_80_NO_12_P | 820.5253 | [M+Na]^+^ | 7.38 | 4.33↑ | 6.65E-08*** | | 6.3108E-05 | Sphingolipids-Ceramide phosphoinositols |
| 91 | 11β-hydroxyandrost-4-ene-3,17-dione ^b^ | C_19_H_26_O_3_ | 325.1775 | [M+Na]^+^ | 1.96 | 3.72↑ | 3.13E-07*** | | 4.2220E-04 | Steroids-Androgens |
| 92 | 3-Deoxyvitamin D3^a^ | C_27_H_44_ | 369.3515 | [M+H]^+^ | 6.73 | 1.71↑ | 1.19E-06*** | | 1.6653E-03 | Sterol Lipids-Vitamin D3 |
| 93 | 3α,12α-Dihydroxy-7-oxo-5β-cholestan-26-oic acid ^b^ | C_27_H_44_O_5_ | 471.3065 | [M+Na]^+^ | 0.88 | 3.77↑ | 5.63E-07*** | | 8.2203E-04 | Sterols-Cholesterol and derivatives |

^a.^ Metabolites validated based on metlin database. ^b.^ Metabolites putatively annotated. ^c.^ Metabolites validated based on their fragment ions.

(↑): up-regulated and (↓): down-regulated. **p*<0.05, ***p*<0.01, ****p*<0.001 versus Sham.


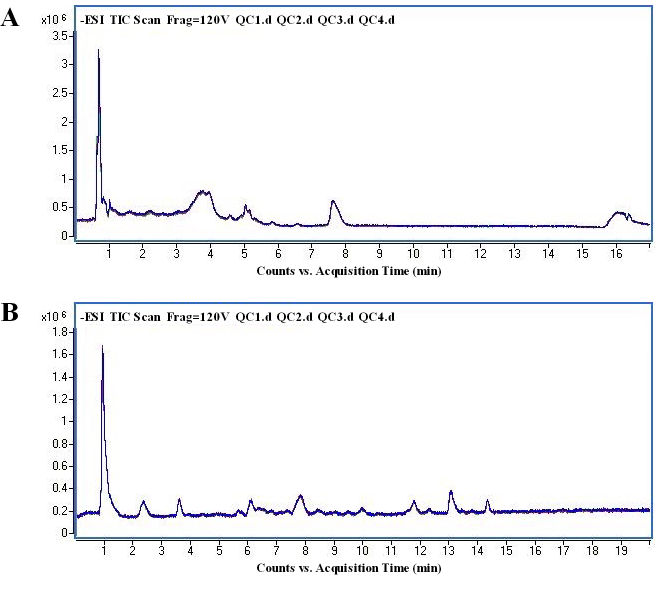
**Figure S1** The overlapped total ion chromatography (TIC) of 4 QC samples in ESI negative ion mode in both metabolomic analysis (**A**) and lipidomic analysis (**B**).


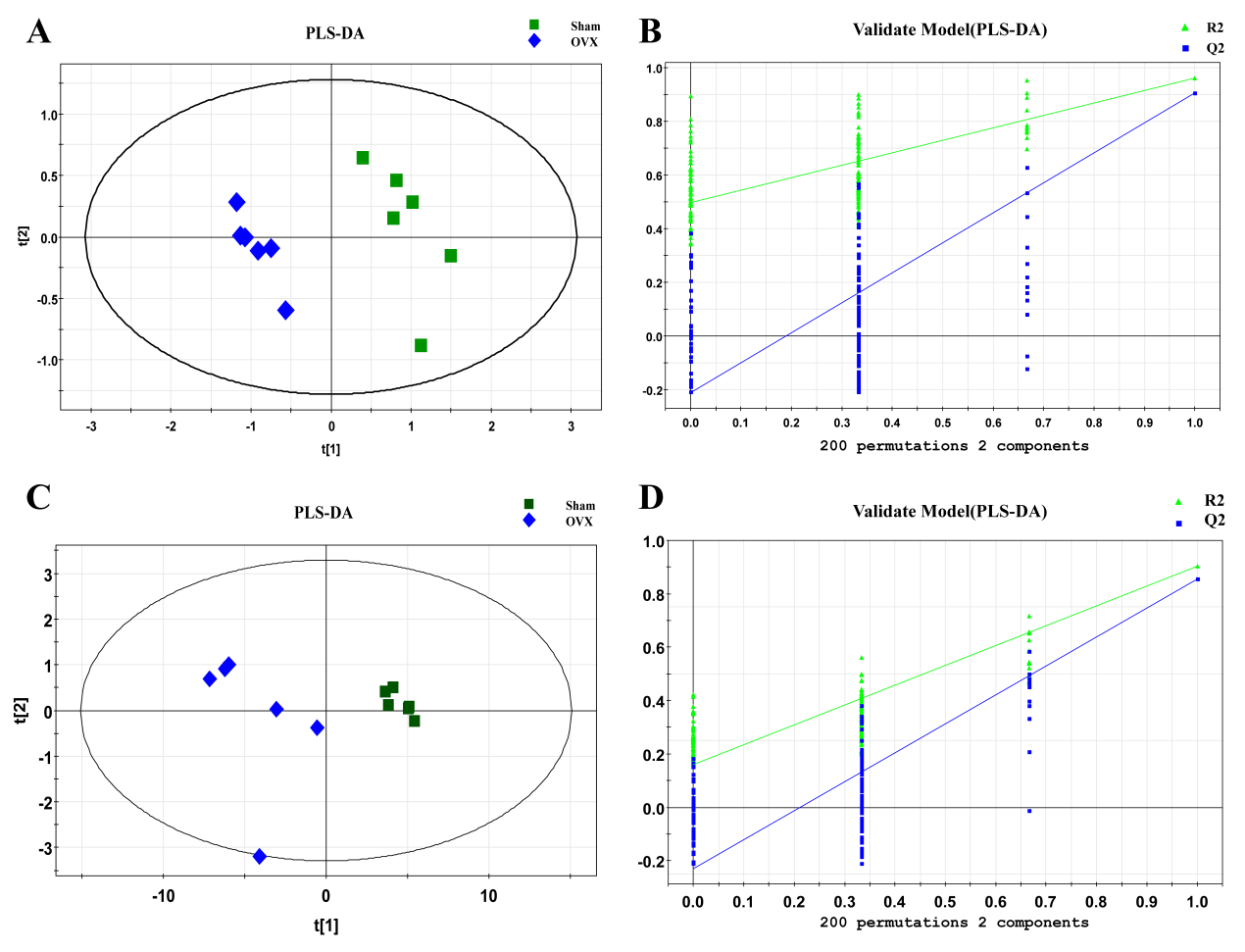


**Figure S2**  Plots of multivariate statistical analysis of the experimental group in ESI negative ion mode.**(A, C)** PLS-DA score plot of the sham and OVX group in metabolomic and lipidomic analysis respectively; **(B,D)** Permutation test plot of the sham and OVX group in metabolomic and lipidomic analysis respectively.


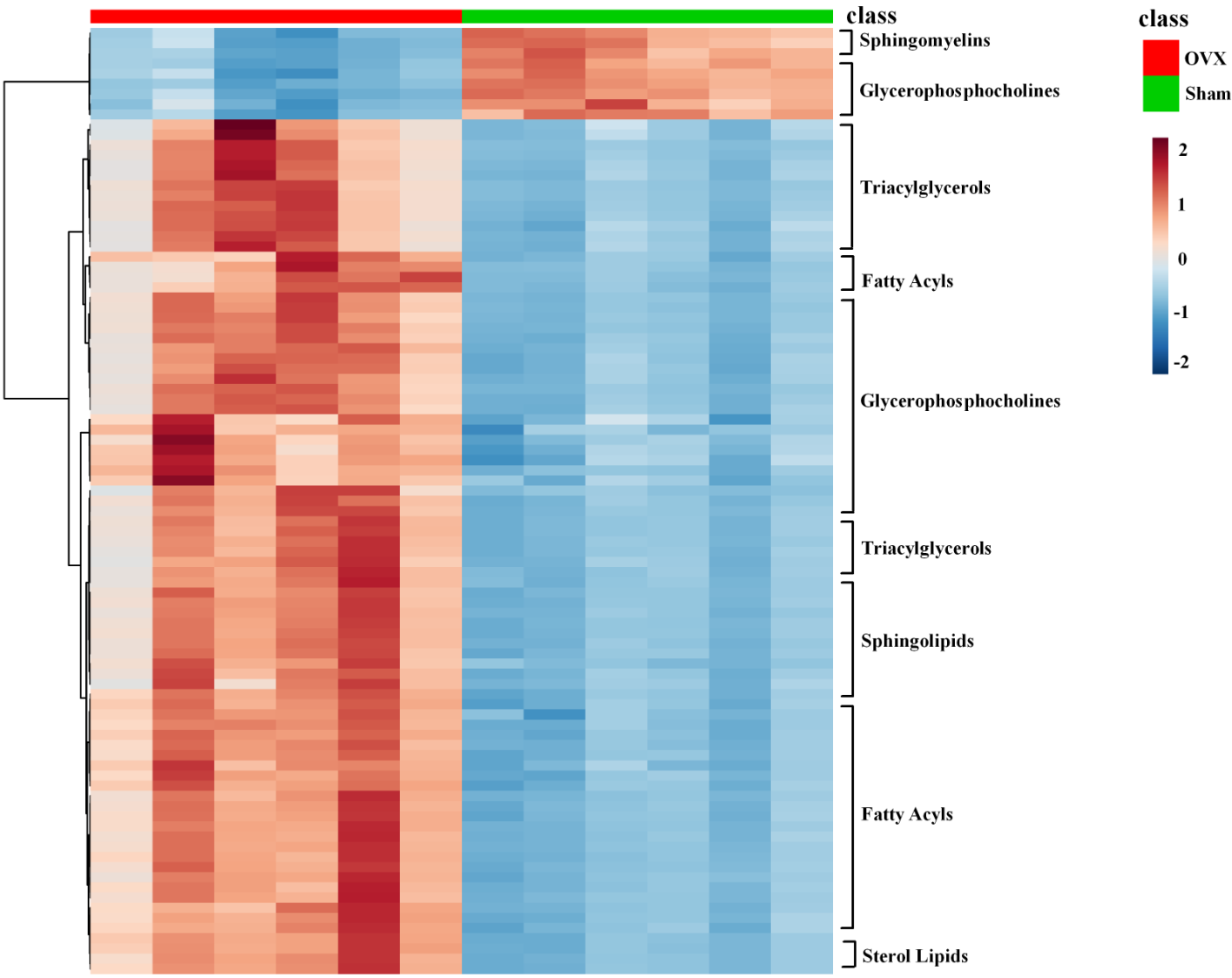


**Figure S3** Heat map based on the relative levels of potential marker lipids in femur of mouse in OVX. Color key indicates metabolite expression value, red: up-regulated, blue: down-regulated.


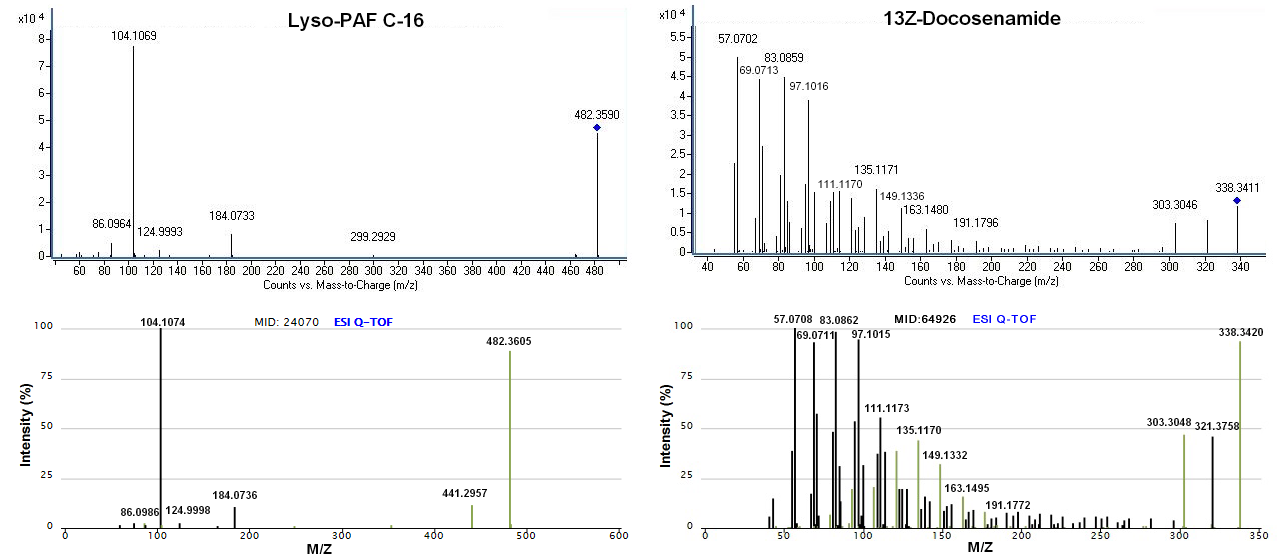


**Figure S4** Lipids identification process based on metlin database. The upper panels were the product ion spectrum of potential biomarkers in our experiments; the lower panels were the data in metlin database oneline.

**
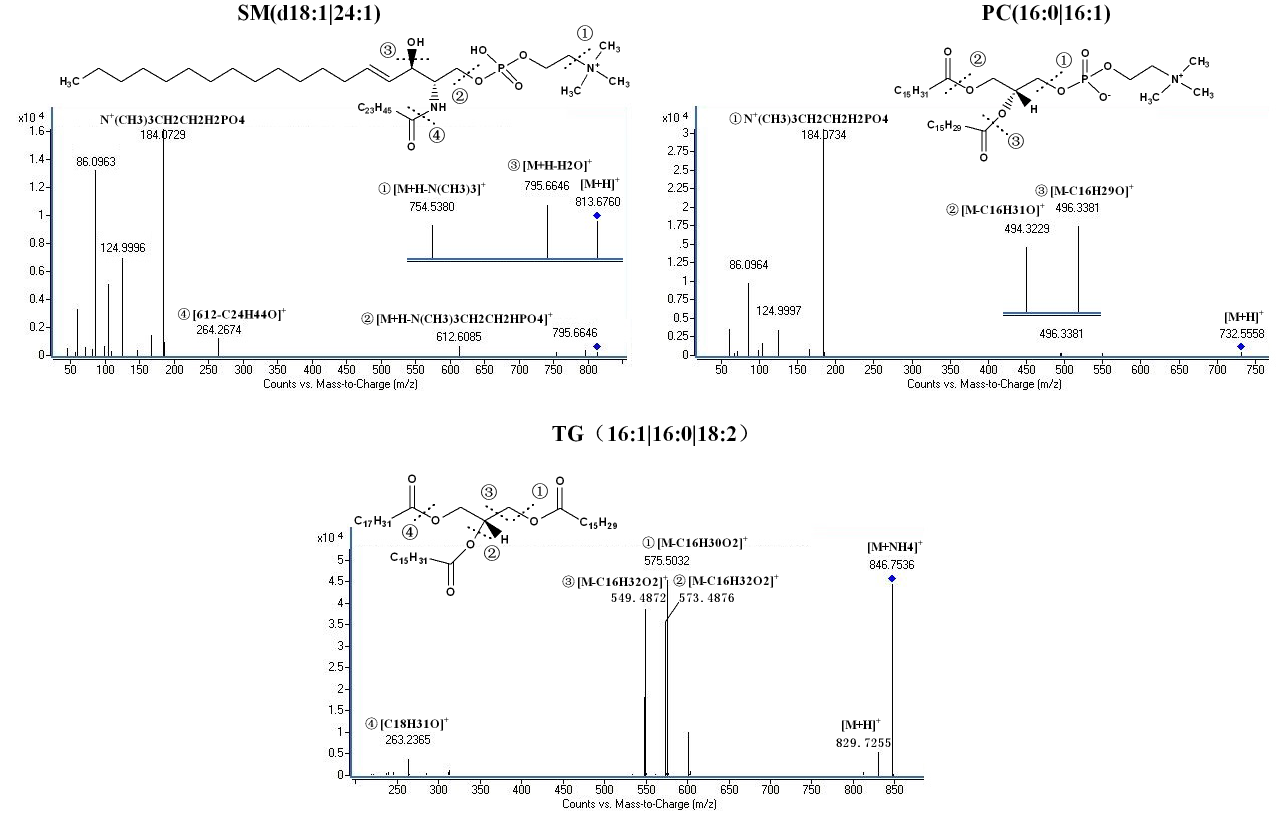
**

**Figure S5** Lipids identification process based on characteristic molecular ion information and corresponding fragments of product ion.
